# No-dependent signaling pathways in unloaded skeletal muscle

**DOI:** 10.3389/fphys.2015.00298

**Published:** 2015-10-31

**Authors:** Boris S. Shenkman, Tatiana L. Nemirovskaya, Yulia N. Lomonosova

**Affiliations:** ^1^Institute of Biomedical Problems, Russian Academy of SciencesMoscow, Russia; ^2^Faculty of Fundamental Medicine, Lomonosov Moscow State UniversityMoscow, Russia

**Keywords:** skeletal muscle, unloading, nitric oxide (II), NO-synthase, protective function of NO

## Abstract

The main focus of the current review is the nitric oxide (NO)-mediated signaling mechanism in unloaded skeletal. Review of the published data describing muscles during physical activity and inactivity demonstrates that NO is an essential trigger of signaling processes, which leads to structural and metabolic changes of the muscle fibers. The experiments with modulation of NO-synthase (NOS) activity during muscle unloading demonstrate the ability of an activated enzyme to stabilize degradation processes and prevent development of muscle atrophy. Various forms of muscle mechanical activity, i.e., plantar afferent stimulation, resistive exercise and passive chronic stretch increase the content of neural NOS (nNOS) and thus may facilitate an increase in NO production. Recent studies demonstrate that NO-synthase participates in the regulation of protein and energy metabolism in skeletal muscle by fine-tuning and stabilizing complex signaling systems which regulate protein synthesis and degradation in the fibers of inactive muscle.

Recent studies have highlighted a critical role of NO in the complex signaling regulation in functional muscle fibers. In the present review we summarize role of NO and NOS in anabolic and catabolic signaling processes in skeletal muscle under conditions of elevated and decreased contractile activity.

NO interacts with cell components using three main mechanisms. First, NO reacts with molecular oxygen and superoxide anions, producing a number of low-molecular NO derivatives (Nox) which possess redox activity and are capable of participation in electron transportation reactions (Stamler et al., 1992). The reaction between NO and superoxide anions leads to the production of peroxynitrites, the most reactive free-radical molecules in biological systems which are often used as indicators of oxidative stress (Freeman, 1994). Skeletal muscle produces small amounts of NO and superoxide anions under normal physiological conditions. Secondly, NO derivatives react with metal-transporters such as haem iron and iron-sulfide centers producing NO-metal complexes. NO can also regulate the function of metalloproteins. For example, the NO-haem bond increases soluble guanylate cyclase activity in muscle, raising the concentration of cyclic guanosine monophosphate (cGMP) (Kobzik et al., 1994). Third, reduced thiols are the main targets of NO. NO reacts with thiols of proteins (RSH, RS^−^) through S-nitrosylation. Thus, S-nitrosylation of glutathione and other non-regulatory thiols represents another mechanism of NO buffering by the cells. This reaction can also regulate protein function through conformational changes by increasing disulfide formation, or through reacting with the nearest centers containing metal atoms (Stamler, 1994). Activation of the two main NO-mediated signaling processes depends on guanylate cyclase activation and protein S-nitrosylation.

## Isoforms of NOS and regulation of nNOS activity

Synthesis of NO is catalyzed by NOS. Three NOS isoforms are known: type I or neuronal NOS (nNOS) is continuously expressed in different neuronal structures and in muscles; type II is inducible NOS (iNOS) which usually is not present in muscle cells; type III endothelial NOS (eNOS) is expressed in endothelial cells (Lancaster and Hibbs, 1990; Forstermann et al., 1994). nNOS and eNOS are calcium-dependent, iNOS is calcium-independent and is transcriptionally regulated by different cytokines (Hevel et al., 1991; Stuehr et al., 1991). nNOS and eNOS are regulated by intracellular Ca^2+^ levels through Ca^2+^-dependent binding of NOS to the calcium-calmodulin complex (CaM). nNOS and eNOS initiate activation at a Ca^2+^ concentration of about 100 nmol/L, and are fully activated at 500 nmol/L of Ca^2+^ (Förstermann et al., 1990, 1991; Pollock et al., 1991; Schmidt et al., 1991).

Several isoforms/splice-variants of nNOS are known. nNOSμ is the most well-studied splice variant of nNOS in rat skeletal muscle (Alderton et al., 2001). The main bulk of NO is produced by the nNOSμ isoform in skeletal muscle. It is localized to the subsarcolemmal zone and is associated with dystrophin-sarcoglican complex of cytoskeletal proteins. nNOSμ can also be associated with the cytoskeletal complex at the nuclear membrane (Aquilano et al., 2014). nNOS activity, i.e., its ability to produce NO, is regulated by phosphorylation on the serine residue by insulin-activated protein kinases (Hinchee-Rodriguez et al., 2013) or AMP-dependent protein kinase (AMPK) (Chen et al., 2000). A decrease in NO production is, most likely, caused by calpain-dependent degradation of nNOS or its export out of the subsarcolemmal complex (Lainé and de Montellano, 1998; Crosbie et al., 2002). Heat shock protein 90 (HSP90) plays a critical role in nNOS activation and stability (Bender et al., 1999; Kone et al., 2003; Piech et al., 2003; Papapetropoulos et al., 2004). HSP90 regulates nNOS activity through positive allosteric modulation, leading to the formation of active nNOS conformation or by an increase in nNOS affinity for the calcium-calmodulin complex (CaM) (Song et al., 2001). It was previously reported that nNOS interaction with HSP90 regulates insertion of a haem-group (Minami et al., 1994; Billecke et al., 2002). Blockade of HSP90 causes inhibition of nNOS activity and generation of monomer haem-deficit nNOS which undergoes rapid proteasome degradation (Osawa et al., 2003; Dunbar et al., 2004). Moreover, HSP90 regulates degradation of nNOS by calpain (Averna et al., 2007). NOS substrate, the precursor of NO—L-arginine is reported to be the NOS activator while L-NAME (N-nitro-L-arginine methyl ester hydrochloride) is its competitive inhibitor. Both of these compounds regulate nNOS and eNOS isoforms expressed in skeletal muscle.

## Regulation of the expression and localization of neuronal NOS in skeletal muscle

nNOS is expressed in skeletal muscle fibers and in the axons of motoneurons (Nakane et al., 1993). Immunohistochemistry reveals higher concentrations of nNOS in fast, when compared with slow skeletal muscle fibers (Kobzik et al., 1994). nNOS is usually located near the sarcolemma, and its content is very high at the neuromuscular junctions (Brenman et al., 1996). However, it can also be found in the cytoplasm of muscle fibers as an unbound molecule. In the sarcolemmal zone, nNOS is associated with α1-syntrophin of the dystrophin-sarcoglycan complex (Brenman et al., 1995; Wakayama et al., 1997). This association depends on the presence of thePDZ domain of syntrophin (Adams et al., 2001). Sarcolemmal localization of several other proteins such as aquaporin-4 and mechano-sensitive calcium channel TRPC-1 also depends on the presence of the PDZ domain of syntrophin (Adams et al., 2001; Vandebrouck et al., 2007). nNOSμ, bound to the dystrophin-sarcoglycan complex, was also found inside the inner membrane of the myonuclear envelope (Aquilano et al., 2014).

## Function of NO in a contracting muscle

During increased muscle contractile activity NO production in muscle (Perez et al., 2002; Vassilakopoulos et al., 2003) as well as NO content in blood (with verified muscle origin) is increased (Perez et al., 2002). Rue and colleagues (2007) reported a 48% increase in the NO content during contraction of a single muscle fiber (Pye et al., 2007). This increase of NO content was not observed in the presence of L-NAME, a NOS inhibitor. Zhang et al. (2004) found, that NO is produced in C2C12 skeletal muscle cells during static or dynamic stretching (Zhang et al., 2004). Repeated physical exercises lead to elevated nNOS expression (Tatchum-Talom et al., 2000; McConell et al., 2007). However, the nature of the mechano-dependent nNOS activation still remains unknown. The signaling role of NO during increased muscles contractile activity has not been well studied. L-NAME (NOS inhibitor) prevented a fast-to-slow fiber type transition during chronic electrostimulation of muscles in rats (Martins et al., 2012). This suggests the existence of synergetic effects of NO and calcineurin/NFAT signaling pathways on the regulation of myosin heavy chain expression. Indeed, the authors demonstrated that NO suppresses GSK3β via activation of the guanylate cyclase pathway and that this prevents nuclear export of NFATc1. As a result, increased NO production in the contracting muscle promotes a high level of expression of slow myosin heavy chain isoform.

NO is known to stimulate AMPK (5′-AMP-dependent protein kinase) in skeletal muscle. This increases energy supplies in muscle during aerobic exercise; including biogenesis of mitochondria, activation of β-oxidation of fatty acids, as well as insulin-dependent and insulin-independent glucose transport (Lira et al., 2007, 2010; Deshmukh et al., 2010). Moreover, similar to AMPK, NO is an effective inhibitor of histone deacetylases (Watson and Riccio, 2009) promoting expression of many muscle-specific proteins critical for the contractile activity.

## Roles of NO in activity-dependent muscle hypertrophy

Most of scientists support the idea that two known mechanisms underlying development of activity-dependent muscle hypertrophy are: (1) activation of translation mediated by the protein kinase complex mTORC1, and (2) activation of satellite cell proliferation followed by their differentiation and fusion with neighboring muscle fibers, increasing the number of myonuclei (Bodine, 2006; Bruusgaard et al., 2010). The role of NOS activity in activity-dependent muscle hypertrophy is supported by the muscle compensatory loading in synergist removal experiments. L-NAME administration decreases the level of muscle hypertrophy in these experiments (Zhang et al., 2004). A complete block of the increase in slow myosin heavy chains and α-actin expression was also observed under these conditions (Sellman et al., 2006).

Increased NO production was recently shown to activate signaling pathways promoting mTORC1 activity (Ito et al., 2013). According to the data obtained by several research groups, NOS activation during muscle stretching or resisting exercise initiates proliferation of G_0_-satellite cells (Anderson and Wozniak, 2004; Yamada et al., 2006, 2008; Tatsumi, 2010). Released NO passes through the sarcolemma, and activates hepatocyte growth factor (HGF) through the activity of matrix metalloproteinases. Activated HGF interacts with c-met receptors of satellite cells, promoting their entrance into the cell cycle. Therefore, during resistance exercises NO works as an activator and as a regulator of signaling, and sometimes as a trigger of muscle hypertrophy.

## Regulation of the calpain-mediated cytoskeletal proteolysis

Michetti et al. (1995) described an inhibition of proteolytic activity of μ-calpain of skeletal muscle by iNOS *in vitro*. NO can also prevent multiple effects of calcium ionophores on C2C12 muscle cells, which include degradation of vinculin, intercellular junction proteins, and decrease of total protein content. These effects of NO are mediated by the inhibition of μ-calpain activity via the cysteine S-nytrosylation. Experiments using cell cultures showed that NO also regulates calpain-dependent degradation of cytoskeletal proteins (Koh and Tidball, 2000). Zhang et al. (2004) reproduced these experiments with some modifications. They found that 10% stretching in C2C12 cells increases NO content and nNOS activity, increased synthesis of talin, vinculin, and desmin, and to concomitant fiber stiffness (Zhang et al., 2004). These effects are enhanced by the administration NO precursor L-arginine and by calpain inhibitors, and are blocked by the NO-synthase inhibitor L-NAME. In summary, these data suggest the existence of a significant crosstalk between NOS activity and NO production on one side, and the status of cytoskeletal proteins on the other.

## Gravitational unloading of skeletal muscle: NO production, nNOS content and localization

Experiments using rat tail suspension (Tidball et al., 1998; Suzuki et al., 2007), prolonged bed-rest or immersion of human volunteers (Moukhina et al., 2004; Rudnick et al., 2004) showed that total nNOS content is decreased during gravitational unloading. A decrease of nNOS content in muscle fibers also was observed in mice subjected to weightlessness onboard the International Space Station during a 90-day flight (Sandonà et al., 2012). nNOS mRNA expression was also decreased during simulated gravitational unloading (Lomonosova et al., 2011). We previously reported a decrease of NO levels in rat soleus muscle after 2 weeks of unloading (Lomonosova et al., 2011). This result is in a disagreement with the data from Suzuki et al. (2007) who demonstrated increases in NO in combination with a pronounced decrease of total nNOS content in mouse soleus muscle after 14 days of tail suspension. At the same time, levels of NO in nNOS knockout mice and in mice treated with nNOS inhibitor were the same as in control wild type mice (Suzuki et al., 2007). Suzuki and colleagues explained the increase of NO concentration in muscle fibers in their experiments by a translocation of NOS from the membrane to the cytoplasm. This phenomenon was observed on multiple occasions, but currently there are no published data documenting higher NO production by non-membrane-bound NOS. Since both studies (Suzuki et al., 2007; Lomonosova et al., 2011) used the same experimental approach measuring of NO levels the observed discrepancies need further examination.

Sarcolemmal nNOS content was recently shown to be decreased during gravitational unloading (Sandonà et al., 2012). This was associated with protein degradation and with translocation of a part of the nNOS to the cytosolic compartment (Suzuki et al., 2007; Sandonà et al., 2012; Sun et al., 2013; Liu et al., 2014). Lawler and colleagues demonstrated, that administration of EUK-134 blocked nNOS dissociation from the sarcolemma and its translocation to the cytosolic compartment during 54-h unloading experiments (Lawler et al., 2014). This protected pFOXO3a (phospho-Forkhead transcription factor) from de-phosphorylation, and as a result prevented atrophy of soleus muscle and slow-to-fast shift in muscle fiber type (Lawler et al., 2014). These effects could be attributed to prevention of the decrease of total nNOS content due to the changes in nNOS activity related to its release from the sarcolemma. Moreover, nNOS is translocated not only to the cytoplasm, but also to the nucleus along with α-syntrophin (Aquilano et al., 2014). It was noted that altered nNOS splicing and nuclear localization could be a contributing factor in pathology of muscular diseases in humans. Data from Blottner's group demonstrated that after prolonged hypokinesia, not only did nNOS content decrease but also total levels of protein nitrosylation were diminished in human soleus and vastus lateralis muscles (Salanova et al., 2008).

## Gravitational unloading of skeletal muscle: Effects of NO on degradation of cytoskeletal proteins

Unloading leads to the destruction of cytoskeletal and contractile proteins. These changes are usually attributed to increased activity of calcium-dependent proteases calpains. Increased concentration of calcium ions in the sarcoplasm was recently demonstrated at the early stages of gravitational unloading (Ingalls et al., 1999; Shenkman and Nemirovskaya, 2008) along with increased expression of μ-calpain and decreased expression of calpostatin, an endogenous inhibitor of calpains (Ma et al., 2011; Lomonosova et al., 2012b). Yet, Tidball's group (Samengo et al., 2012) found sufficient content of calpain in muscle fibers of young mice to support active proteolysis. In their experiments calpain molecules in muscles of young mice were nitrosylated and did not possess their typical level of proteolytic activity. To the contrary, old animals had lowered levels of nitrosylation, correlated with increased calpain proteolytic activity and decreased content of cytoskeletal proteins. The described experiments clarify possible mechanisms of calpain activation during conditions of decreased nNOS activity.

**Figure 1:**
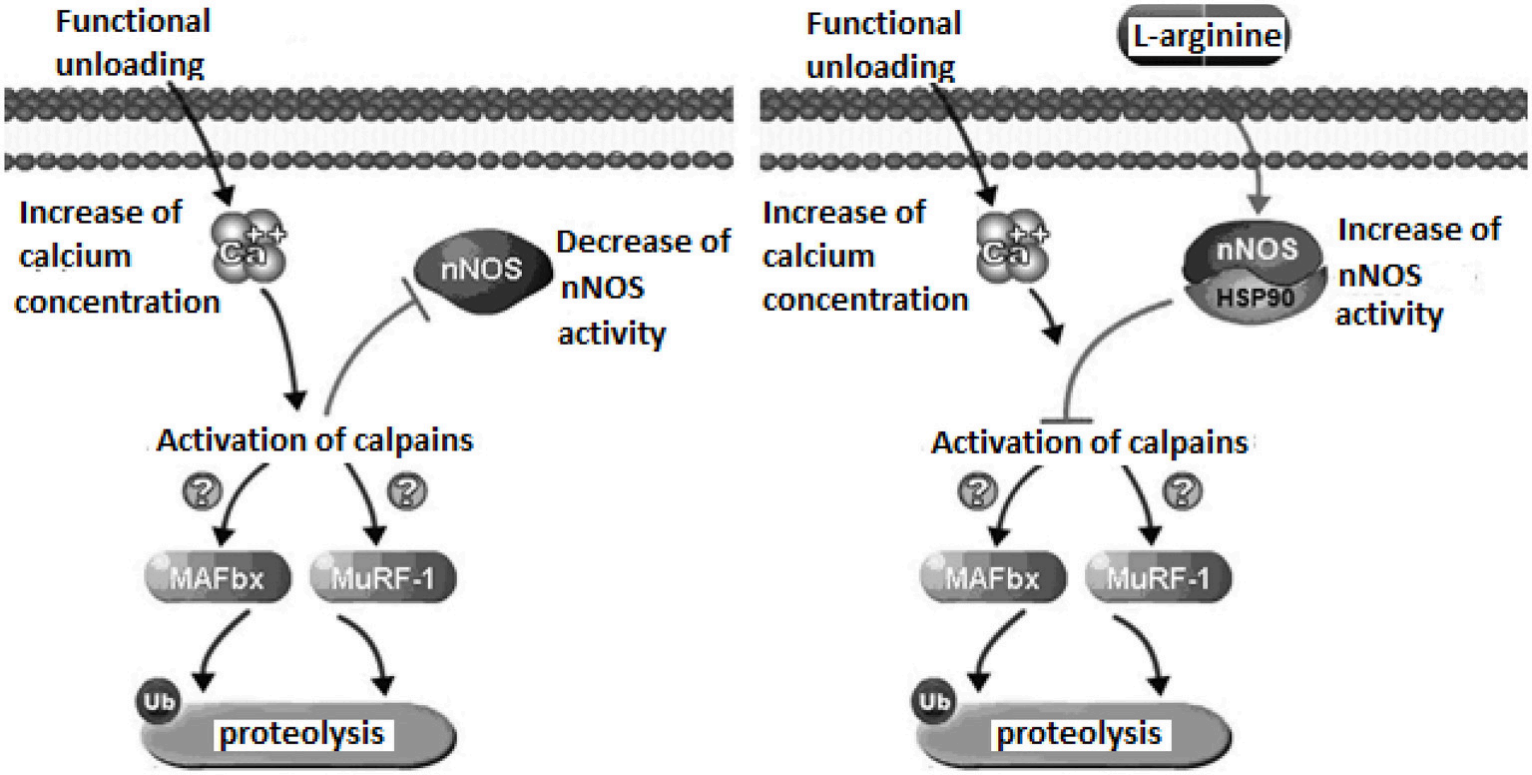
**Mechanismof nNOS effects on protein metabolism in skeletal muscle during unloading**.

Our experiments with administration of the natural NO precursor L-arginine demonstrated prevention of calpain-dependent hydrolysis of cytoskeletal proteins during unloading. In Figure 1, we presented the proposed mechanism of nNOS-induced effects on protein degradation in skeletal muscle during unloading. We hypothesize that lowered NO concentration in muscle during unloading can cause reduced S-nitrosylation of calpains. This leads to calpain activation and to the degradation of cytoskeletal and contractile proteins, resulting in muscle atrophy. To test our hypothesis we administered L-arginine to rats during unloading experiments. Two weeks of unloading decreased NO concentration in muscle, while L-arginine administration prevented any NO decrease and diminished atrophy of soleus muscle. Moreover, L-arginine supplementation prevented the decrease of several cytoskeletal proteins (desmin and dystrophin) in rat soleus muscle during unloading (Lomonosova et al., 2011). Administration of the NO precursor also prevented an increase in the mRNA expression levels of E3-ubiquitin ligases (atrogin-1/MAFbx, MuRF-1—muscle RING-finger protein-1) during unloading.

Recent experiments demonstrated a role for NO as an endogenous inhibitor of 26S proteasomes (Liu et al., 2014). The main function of the proteasome is to degrade unnecessary or damaged proteins by proteolysis. Therefore, lack of increase in the expression of E3-ubiquitin ligases after administration of L-arginine during unloading can be related to a decrease of calpain activity. There are several explanations for the increased expression of ubiquitine ligases due to the activation of μ-calpain (Smith and Dodd, 2007) and calpain P94 (Kramerova et al., 2005). nNOS activity can be also modulated through crosstalk with other regulatory proteins, including heat shock protein 90 (HSP90). Interaction with HSP90 increases nNOS activity and protects cytoskeletal proteins from ubiquitin proteasome degradation (Peng et al., 2009). Under normal conditions skeletal muscle contains high amounts of HSP90 proteins which become significantly decreased during skeletal muscle atrophy (Sakurai et al., 2005; Sõti et al., 2005; Seo et al., 2006). In our study on the administration NO precursor L-arginine during unloading we have not observed a loss of HSP90 content in skeletal muscles of rats administered L-arginine (Lomonosova et al., 2011). Therefore, the decrease of soleus muscle atrophy in our experiments could be in part, the result of HSP90 protection of muscle proteins from ubiquitin proteasome degradation. Thus, activation of NO-dependent pathways can inhibit the signaling systems regulating protein degradation in muscle during unloading, supporting a critical role of NO signaling in these conditions.

## Gravitational unloading of skeletal muscle: Effects of NO on fiber type composition

Unloading is known to stimulate expression of the fast isoforms of myosin heavy chains (MHC) leading to changes in contractile properties of soleus muscle. Unexpectedly, in our study we found that L-arginine administration also prevented a decrease of slow MHC expression levels in soleus muscle during unloading (Lomonosova et al., 2011). This data demonstrates a critical role of nNOS in the regulation of contractile protein expression. Based on the data on inhibition of the calcineurin/NFAT by GSK3β and its regulation by the NO-induced cyclic guanosine monophosphate pathway (Martins et al., 2012), we hypothesized that increases in NO content after L-arginine administration in our experiments prevents slow-to-fast transition in soleus muscles of rats.

## Gravitational unloading of skeletal muscle: Effects of mechanical activities on nNOS

Most of scientists link alterations in skeletal muscles following unloading with a lack of muscle contractile activity or with decreased resistive components of muscle contraction (Falempin and Mounier, 1998). The same can be said about changes in the NO-dependent signaling pathways. Interestingly, mechanical stimulation of a plantar supporting zone with concurrent increases of electric activity of soleus (Grigoriev et al., 2004) prevents a loss of nNOS during immersion experiments. These data demonstrate the dependence of NOS content on muscle contractile activity (Moukhina et al., 2004). Resistance physical exercise during hypokinesia also blocks decreases of protein nitrosylation levels (Rudnick et al., 2004). Therefore, decreased muscle contractile activity leads to a loss of nNOS content and activity. Chronic passive stretching is often used to simulate the loading component of muscle contraction. We hypothesized that nNOS can participate in atrophy prevention in soleus muscle under conditions of stretching combined with rat tail suspension.

To test our hypothesis, we blocked NOS with L-NAME during stretching combined with unloading (Lomonosova et al., 2012a). Muscle membrane stretching induces nNOS activity (Deshmukh et al., 2010) preventing an increase in the activity of calpains, expression of E3-ubiqutin ligases, and protein ubiquitination during unloading. The content of nNOS is not decreased during stretching combined with unloading (Lomonosova et al., 2012a; Xu et al., 2012). If our hypothesis is correct, administration of the nNOS blocker L-NAME during stretching, combined with unloading would result in muscle atrophy. However, in our study, NOS inhibition in stretched soleus muscle during rat tail suspension does not cause muscle atrophy. In fact, the level of E3-ubiquitin ligases was not increased in any of the tail suspended rats in our experiments with stretched soleus muscle with or without inhibition of NOS activity. We concluded that NO-dependent signaling is not critical for the maintenance of muscle mass in stretched soleus muscle during unloading. At the same time, muscle stretching during unloading completely prevented the decrease of slow and increase of fast MHC expression (Falempin and Mounier, 1998). L-NAME administration neutralized this effect of stretching, supporting a role of NO in preserving MHC expression during muscle stretching, combined with unloading.

Some authors have linked increased muscle mass with the higher content of myonuclei and activation of the satellite cells in muscle (Wang et al., 2006; Shenkman et al., 2010). A decrease in NO concentration was previously reported to lower the ability of satellite cells to proliferate. An increase in the number of satellite cells was prevented by administration of L-NAME in stretched muscle (Shenkman et al., 2010). This suggests that satellite cell activation and proliferation is a NO-dependent process (Kartashkina et al., 2010).

## Conclusion

Exogenous activation of nNOS and probably other NOS isoforms leads to an increase in NO production. This can block damage and atrophic processes in skeletal muscle, as well as changes in the expression of myosin isoforms under unloading conditions. The expression and content of NOS is decreased during unloading and this coincides with decrease in NO production. Based on these findings, we hypothesize that a reduction of NOS and NO production promotes muscle atrophy. Our hypothesis is supported by recent studies demonstrating that NO-synthase participates in the regulation of protein turnover in skeletal muscle by fine-tuning and stabilizing signaling pathways that regulate protein synthesis and degradation in the muscle fibers of inactive muscle (Table 1).

**Table 1:** **NO-related responses in active and unloaded muscle**.

	**Active muscle**	**Ref.**	**Unloaded muscle**	**Ref.**
NO content	High	36,37, 38	Low	62
nNOS content	High	40,41	Low	57–66
NO inhibits calpain-induced protein degradation	Yes	47,56	No	62
NO maintains slow myosin phenotype	Yes	42	No	62
NO maintains satellite cells proliferation	Yes	51–54	No	82–84

## Funding

The work was supported by RFBR grants 13-04-00888, 11-04-00788, 14-04-01632.

### Conflict of interest statement

The authors declare that the research was conducted in the absence of any commercial or financial relationships that could be construed as a potential conflict of interest.
